# Repetitive transcranial magnetic stimulation and constraint-induced movement therapy combined in the treatment of post-stroke movement disorders: a narrative review

**DOI:** 10.3389/fnhum.2025.1578258

**Published:** 2025-04-07

**Authors:** Zhennan Liu, Qingying Yu, Feng Zhou, Muyao Yu, Huan Shu, Manhua Zhu, Tianzhong Peng

**Affiliations:** ^1^Graduate School of Jiangxi University of Traditional Chinese Medicine, Nanchang, China; ^2^Department of Rehabilitation Medicine, Hongdu Hospital of Traditional Chinese Medicine, Nanchang, China

**Keywords:** post-stroke dyskinesia, repetitive transcranial magnetic stimulation, constraint-induced movement therapy, combination therapy, review

## Abstract

Stroke is a significant cardiovascular and cerebrovascular condition and is among the primary causes of prolonged neurological impairment globally. Approximately 55%–75% of stroke survivors will experience some form of long-term sensorimotor impairment. Post-stroke, the upper limb typically exhibits restricted mobility, complicating daily chores for 70% of patients and impairing normal limb utilization. Repetitive Transcranial Magnetic Stimulation (rTMS), a prominent non-invasive neuromodulation technique designed to enhance functional recovery post-stroke, has garnered significant attention in clinical studies. Likewise, constraint-induced movement therapy (CIMT) has been extensively employed in therapeutic settings to promote neuroplasticity. However, there remain several issues with it in practical application. Recently, considerable focus has been directed toward a novel treatment known as rTMS in conjunction with obligatory motor therapy. This can circumvent the issues associated with conventional treatments and optimize the advantages of both. This article discusses the present status of clinical research with rTMS and CIMT.

## 1 Introduction

Stroke, a significant cerebrovascular disorder, is typically induced by the rupture or obstruction of blood arteries, leading to impairment of cerebral function and disruption of regulatory mechanisms. The prevalence of this disease in China is comparatively elevated on a global scale, making it one of the primary contributors to mortality and disability among adults (Wang, 2021). Common functional deficits following a stroke include motor impairments, sensory anomalies, speech difficulties, discomfort, and restricted capacity to perform activities of daily living (ADLs). Patients may experience mobility challenges, which might complicate their everyday activities and significantly diminish their quality of life (Ruan and Ye, 2024). Researchers have mostly examined the efficacy of either CIMT or rTMS in facilitating the restoration of normal bodily function in stroke patients (Abdullahi et al., 2023), few research examine the synergistic effects of constraint-induced mobility therapy (Duan et al., 2024). Prior studies have mostly examined the efficacy of constraint-induced movement therapy and rTMS in aiding individuals with motor impairment following a stroke. Nonetheless, no extensive research has investigated the synergistic effects of these two therapies in a clinical setting. This article examines studies on the application of repeated transcranial magnetic stimulation in conjunction with constraint-induced movement therapy for the treatment of dyskinesia following a stroke. The objective is to provide readers with an innovative perspective on rehabilitation methods that can assist stroke patients experiencing limb difficulties.

## 2 Repeated transcranial magnetic stimulation

Various forms of magnetic stimulation, namely single-pulse and paired-pulse, have been employed to investigate the reorganization of the brain’s cortex following a stroke. Researchers have discovered that varying stimulation parameters in rTMS can either decrease or increase cortical excitability (Han et al., 2025), which has prompted the development of rTMS therapy. Contemporary mainstream research indicates that the mechanism of action of rTMS in post-stroke dyskinesia may be as follows: Cheng et al. (2023) employed fMRI to investigate the effects of rTMS on brain networks and discovered that it could enhance interhemispheric connectivity by increasing activity in ipsilateral injury regions, augmenting excitatory connectivity from ipsilateral injury to contralateral brain areas, and reducing stroke-induced inhibition across the corpus callosum, thereby facilitating motor performance recovery. Zong et al. (2020) observed that with prolonged rTMS treatment, levels of anti-inflammatory cytokines and mitochondrial MnSOD were elevated in the vicinity of the infarct. This preserved the integrity of the mitochondrial membrane and inhibited the cystatin-9/3 apoptotic pathway occurring spontaneously in the infarcted cortex, hence enhancing the local neuronal microenvironment. The transition of microglia to the M1/M2 phenotype and the shift from A1 to A2 in astrocyte phenotype were associated with accelerated nerve healing facilitated by rTMS. The quantity of neurotrophic factor: Brain-derived neurotrophic factor (BDNF) facilitates neuronal survival, growth, migration, and differentiation, while also enhancing dendritic and axonal growth and synaptic formation following central nervous system injury. Research indicates that the external magnetic field generated by rTMS influences BDNF concentrations in serum and cerebrospinal fluid (Ozkan et al., 2024). Luo et al. (2017) discovered that high-frequency rTMS stimulation elevated blood BDNF levels and its affinity for the TrkB receptor (neuronal receptor tyrosine kinase-2), resulting in increased BDNF and phosphorylated TrkB protein levels. In conclusion, the precise pathways through which rTMS produces its therapeutic effects remain inadequately elucidated, necessitating future research for validation.

Repetitive transcranial magnetic stimulation, a non-invasive and painless neuromodulation technique, is regarded as a treatment that does not harm the internal cranial structures (Klomjai et al., 2015) and is frequently utilized for limb dysfunction, speech loss, visual impairment, swallowing difficulties, motor neurodeficiencies in limbs, and psychiatric disorders following a stroke. Research indicates that over 70% of stroke patients experience varied degrees of motor impairment following the onset of the stroke (Cieza et al., 2020). The effectiveness of rTMS is contingent upon the stimulation frequency, with low-frequency stimulation (≤ 1 Hz) exerting an inhibitory effect and high-frequency stimulation (> 5 Hz) inducing brain excitement (Simonetta-Moreau, 2014). Presently, studies indicate the comparable efficacy of rTMS at 3 Hz and 10 Hz in addressing upper-limb motor dysfunction following a stroke (Xiao et al., 2019). A study conducted by Vabalaite et al. (2021) demonstrated that rTMS at 10 Hz was more effective than low-frequency stimulation on the FMA-UL rating scale. Presently, the majority of individuals believe that low-frequency stimulation is equally effective as high-frequency stimulation for upper extremity rehabilitation (Kim et al., 2014). Two meta-analyses indicate that rTMS enhanced lower limb dyskinesia post-stroke (Ghayour-Najafabadi et al., 2019) and significantly augmented walking speed (Li et al., 2018). Numerous studies have validated the efficacy of rTMS in treating lower limb dyskinesia. The ideal therapeutic target of rTMS remains ambiguous, necessitating further research to assess and quantify the various stroke phases of the pathological substrates, thereby determiningat the exact neural grid localization at the dorsolateral prefrontal cortex to find the optimal therapeutic target. It involves focal magnetic stimulation applied externally to the scalp, typically at the dorsolateral prefrontal cortex, which induces electrical stimulation in underlying cortical tissue (Cash et al., 2021). High-frequency magnetic stimulation, rTMS, alters the excitability of cortical neurons, such as those in M1. This is what enables neuroplasticity in the brain (Chen et al., 2024). Constraint-Induced Movement Therapy (CIMT) seeks to transform the activation of brain pathways into functional motor performance. By compelling the impacted limb to engage in high-intensity task training, we convert neural remodeling into tangible motor improvements (Zhang et al., 2024).

## 3 Constraint-induced movement therapy

Constraint-induced movement therapy is a well-established rehabilitation program that is crucial for addressing limb motor impairment in stroke patients. Taub (1976) indicated research indicates that primate trials reveal that severing somatosensory afferent nerves leads to a phenomenon of learnt disuse, subsequently constraint-induced movement therapy. Researchers have conducted comprehensive studies from various perspectives and identified several potential mechanisms of action: The α-amino-3-hydroxy-5-methyl-4-isoxazolepropionic acid receptor (AMPAR) serves as the principal ionotropic glutamate receptor (GluR) inside the central nervous system of animals. AMPARs are located in cortical neurons and hippocampus pyramidal neurons, primarily consisting of GluR1/2 and GluR3/4 heterodimers. As noted by Hu et al. (2020), CIMT therapy resulted in an augmentation of GluR2-containing synapses and an elevation of GluR2 expression in the ipsilateral sensorimotor cortex synapses. rTMS particularly high-frequency stimulation, might enhance the aggregation of NMDAR receptors at the postsynaptic membrane by causing long-term potentiation (Kweon et al., 2024), with both mechanisms potentially complementing each other in the context of synaptic plasticity. CIMT enhances synaptic connection in behavioral training, whereas rTMS directly alters the synaptic protein production pathway via electromagnetic stimulation. The CST (corticospinal tract), a significant white matter bundle comprising the pyramidal tract, internal capsule, and cerebral tegmentum, functions as a primary neuronal conduit for voluntary movement. Hu et al. (2019) examined the anisotropy scores and mean diffusion coefficients of the CST in wind mice before and after CIMT using diffusion tensor imaging, concluding that CIMT enhances functional recovery post-ischemic stroke by aiding the rebuilding of the ipsilateral CST. rTMS also elevates cerebral blood flow in the targeted region and ameliorates hypoxia within the CST milieu (Heejae, 2024), these effects may synergistically promote axon guidance and myelin regeneration in the CST. A study utilizing bioinformatics prediction and luciferase assays discovered that CIMT may enhance neuronal and myelin remodeling in the motor cortex by partially inhibiting the c-Jun/miR-182-5p/Nogo-A pathway. This enhances the capacity for comprehension and equilibrium (Tang et al., 2023). Researchers have identified that rTMS directly suppresses the promoter activity of Nogo-A and stimulates the cAMP-PKA-CREB pathway (Bao et al., 2021). Two distinct inhibitory actions may occur simultaneously: CIMT reduces Nogo-A post-transcriptionally, while rTMS inhibits its synthesis during transcription. While the role of CIMT in stroke patients has been evidenced in multiple investigations, the specific mechanism by which it has a predominant effect remains unestablished, necessitating future research for clarification.

It aims to enhance movement abnormalities by promoting the utilization of limbs with compromised motor function following a stroke. Constraint-induced movement therapy has demonstrated significant efficacy and is frequently employed as a standard protocol in stroke rehabilitation (Shen et al., 2019). Frequently employed as a directive protocol in stroke therapy exercises, it has shown considerable effectiveness. To investigate enhancements in lower limb rehabilitation, Aloraini (2022) conducted a randomized controlled, single-blind clinical investigation involving 38 individuals experiencing dyskinesia post-stroke. The patients were randomly allocated to the CIMT group or the control group. In comparison to the control group, patients in the CIMT group exhibited enhanced recovery of lower limb mobility, postural stability, and gait velocity, with these advancements maintained 3 months following the conclusion of the treatment regimen. 3 months post-treatment, these enhancements persisted; numerous researchers have investigated CIMT therapy in upper limb rehabilitation. Thirty cases of persistent hemiplegia patients who had CIMT therapy have been included. The treatment group exhibited a significant enhancement in upper limb function relative to the control group (Rocha et al., 2021). It can be seen that both CIMT therapies can effectively promote the rehabilitation of upper and lower limb motor functions.

## 4 Repetitive transcranial magnetic stimulation combined with constraint-induced movement therapy

### 4.1 Potential mechanisms

Conventional stroke therapy methods primarily emphasize peripheral rehabilitation tactics to facilitate the relearning and recovery of motor functions. Nonetheless, these techniques exhibit issues including prolonged rehabilitation duration, restricted clinical efficacy, and elevated therapy stagnation rates, with certain patients demonstrating minimal progress in motor dysfunction or remaining at baseline for extended periods (French et al., 2016; Legg et al., 2017). The research commenced with closed-loop rehabilitation theory and then transitioned to the central-peripheral synergistic intervention paradigm. This methodology directly addresses brain injury via “top-down” neuromodulation and integrates it with peripheral functional training to establish a bidirectional closed-loop intervention (Jia, 2016). Non-invasive neuromodulation techniques, including transcranial direct current stimulation (tDCS), rTMS, and mirror therapy (MT), are employed in central interventions to stimulate the neuroplasticity of the motor cortex and reconstruct neural network connections. In contrast, peripheral interventions incorporate CIMT and additional strengthening training tools to facilitate functional remodeling of the limbs through task-oriented training. The synergistic effect may arise from the optimization of limb movement through central intervention and the enhancement of behavioral output via peripheral intervention, thereby transcending the constraints of singular therapy and offering a more focused intervention strategy for stroke rehabilitation (Yan, 2021).

Despite both rTMS and CIMT being grounded in neuroplasticity, they exhibit substantial distinctions in their mechanisms and intervention targets. rTMS employs electromagnetic pulses to directly activate the cerebral cortex. It regulates neuronal firing and alters the functionality of neural networks at the central level (Kwakkel et al., 2015; Lefaucheur et al., 2017). CIMT alters brain function by engaging peripheral behavioral interventions and motor feedback pathways. This reverses the phenomenon of “learned disuse”. Due to the modifiable parameters, rTMS is applicable at all phases of brain injury, ranging from acute to chronic (Zhou et al., 2023). CIMT requires patients to possess fundamental motor abilities and is primarily utilized during the chronic or functional plateau phase. The disparity in mechanisms may stem from rTMS directly influencing central electrical activity to disrupt neural inhibition in pathological conditions, whereas CIMT enhances motor learning via peripheral training, targeting the deficiencies within the “central-peripheral” bidirectional control system. In the initial trial, rTMS was administered exclusively while the individuals were at rest. The impacts of various activities occurring during, before to, and after to the stimulation were disregarded. The relationship between physical training and rTMS remains inadequately investigated in the existing literature (Beaulieu and Milot, 2018). Clarifying the link between the two is crucial for investigating the CNS adaptation mechanism. CIMT promotes motor function compensation and neuronal reconfiguration via behavioral interventions by restricting training on the unaffected side of the limb and enhancing it on the affected side. This leads to both functional compensation and brain remodeling. Research has revealed that rTMS applied during prolonged voluntary tonic activity enhances the synchronization of motor neurons. The integration of behavioral stimulation enhances neuronal plasticity in the motor cortex beyond that achieved by rTMS alone and assists rTMS treatments in determining the optimal mix of intensity, duration, frequency, and location (Mills and Schubert, 1995). Furthermore, in terms of neurotrophic factors, according to Livingston-Thomas, the mechanism of action of CIMT in the restoration of motor function in the upper limb may be closely related to its modulation of microglial cell function: although post-stroke rehabilitation did not alter the total proportion of BDNF-expressing cells in the infarcted area, it was found, by immunofluorescence assay, that CIMT induced a significant increase in the proportion of non-neuronal/non-astrocyte-derived BDNF was significantly increased, and a functional transformation of the subpopulation of BDNF-expressing microglia was also observed (Livingston-Thomas et al., 2014), the cellular phenotype may have shifted from a pro-inflammatory M1-type to a reparative M2-type, and this phenotypic polarization may have occurred either through inhibition of TAK1 -NF-κB-p38 MAPK signaling pathway, thereby attenuating neuroinflammation and enhancing tissue repair (Vinnakota et al., 2024). This technique exhibits synergistic potential with high-frequency rTMS, which facilitates neurological recovery by stimulating the BDNF/TrkB signaling pathway, so establishing a closed-loop “central-peripheral-central” regulation (Luo et al., 2017). Consequently, the synergistic mechanism of rTMS may involve the enhancement of central neuroplasticity by modulating motor cortex excitability and the BDNF signaling pathway, while simultaneously inhibiting the inflammatory signaling pathway to facilitate the transformation of microglia to the M2 phenotype. In contrast, CIMT activates sensory-motor feedback at the peripheral level through intensive exercise training, thereby further reinforcing central remodeling and sustaining the reparative phenotype of microglia. This multimodal intervention produces a compounded effect across the three aspects of neuroinflammation management, neurotrophic factor release, and synaptic plasticity repair, hence overcoming the limitations of single therapy efficacy.

Synergistic potential of rTMS paired with CIMT in stroke recovery, numerous problems persist within this program: Attention must be given to potential safety concerns and parameter optimization, such as the risk of electromagnetic interference between TMS and implanted devices (Rossi et al., 2021). Additionally, excessive use of CIMT may lead to muscle strain in the training limb or psychological resistance in patients, thereby increasing risk. The therapeutic synergies, specifically the temporal alignment between the neuromodulation after-effect window (approximately 60 min) of rTMS and the intensity of CIMT training, remain unclear. Furthermore, individual adaptability is a concern, as patients with severe motor disorders (Fugl-M) may experience central fatigue due to over-stimulation. The relationship between the therapeutic synergy of rTMS’s neuromodulation after-effect window (about 30–60 min) and the intensity of CIMT training remains ambiguous, and excessive stimulation may lead to central weariness (Zhu et al., 2023). Individuals with significant movement problems (Fugl-Meyer score < 30) may lack the capacity for effective CIMT, hence diminishing the treatment’s efficacy. The combination treatment needs two and a half to three hours daily, incurs significant costs, necessitates extensive equipment, and consequently poses challenges for clinical promotion. Stringent requirements provide obstacles to clinical dissemination (Sharma et al., 2023). Addressing these issues necessitates enhanced patient classification, adaptive parameter adjustment mechanisms, and validation through multicenter, large-scale research.

### 4.2 Application and progress

**FIGURE 1:**
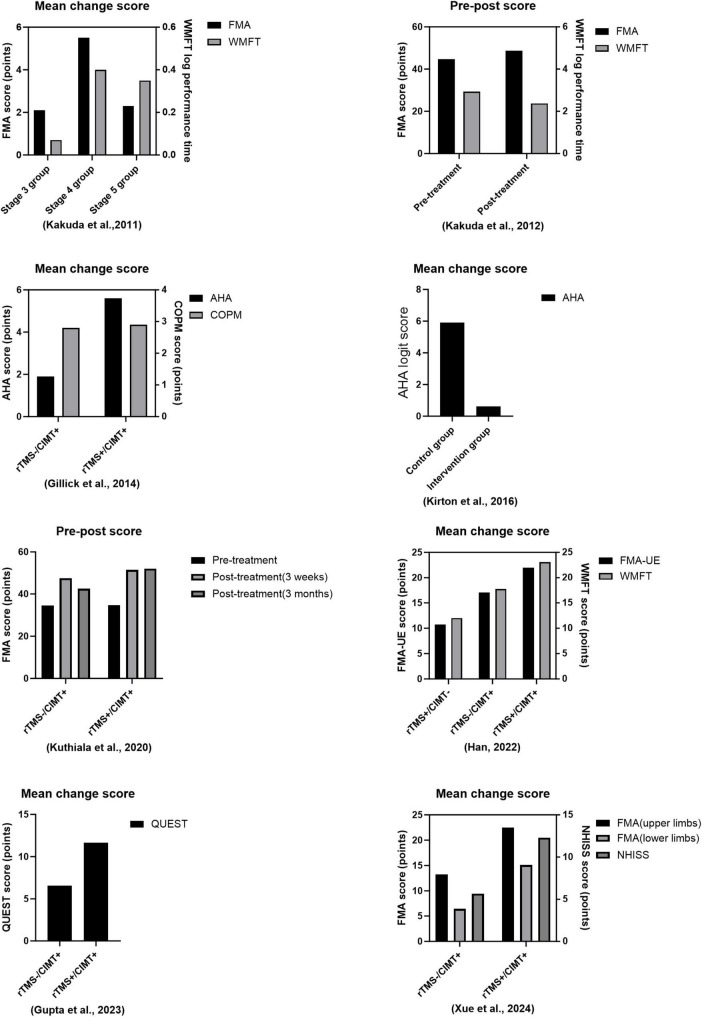
Comparison of efficacy of combination therapies.

Table 1 and Figure 1 give more details of relevant combination therapies studies, so only more critical points are discussed here. A study including 52 stroke patients with upper limb hemiparesis shown that a 15 days treatment utilizing a combined protocol of CIMT and 1 Hz rTMS to stimulate the contralesional hemisphere was effective. The results indicated a substantial correlation between the extent of improvement in the Fugl-Meyer and Wolf Motor Function Test (WMFT) and the initial Brunnstrom stage, implying that the intervention’s efficacy is contingent upon the initial severity of hemiparesis, thereby affirming the safety of the combination therapy and offering an evidence-based foundation for the formulation of personalized rehabilitation strategies (Kakuda et al., 2011). Kakuda et al. (2012) conducted a follow-up study examining the efficacy of 1 Hz rTMS in conjunction with CIMT in individuals with upper extremity hemiparesis post-stroke. They aimed to direct rTMS stimulation to the contralesional hemisphere above the major motor region for a duration of 15 days. The findings indicated that the patients’ motor function score for the upper extremity (median FMA score increased from 47 to 51) and motor efficiency (median WMFT log performance time decreased from 3.23 to 2.51) exhibited significant improvement at discharge compared to baseline levels at admission, thereby affirming the clinical efficacy of the combination protocol in enhancing limb motor control and functional recovery. Hemiplegic cerebral palsy is a prevalent subtype typically caused by a prenatal stroke. Perinatal strokes exemplify how human development can evolve over time, as they occur at specific intervals in healthy brains (Peter et al., 2007). To elucidate the feasibility and efficacy of rTMS in conjunction with CIMT for the rehabilitation of paralyzed hands in children with cerebral palsy (CP), Gillick et al. (2014) stratified 19 children aged 8–17 years into two cohorts, each undergoing five consecutive sessions of either real or sham rTMS and CIMT alternately, targeting the contralesional hemisphere over the primary motor area for a duration of 14 days. They employed Fisher’s exact test to analyze alterations in individuals’ raw AHA scores with a four-point smallest discernible difference (SDD). The primary measure utilized was the Assisted Hand Assessment (AHA), while the Canadian Occupational Performance Measure (COPM) and Stereognostic indicators served as additional measures. The findings revealed that eight of 10 participants in the rTMS/CIMT group exhibited improvements exceeding the SDD, whereas only two of nine in the sham rTMS/CIMT group demonstrated similar progress. This suggests that low-frequency rTMS combined with CIMT may be safe, feasible, and effective for treating pediatric hemiparesis. It is noteworthy that the rTMS protocol employed by the team was particularly innovative, utilizing a combination of 1 Hz low-frequency and 6 Hz high-frequency alternating stimulation patterns, each with distinct stimuli. The team’s rTMS program is highly novel since it transcends the constraints of single-frequency stimulation modes, such as a solitary 1 Hz, by alternating between 10 min of 1 Hz low-frequency and 6 Hz high-frequency stimulation modes. This dual-frequency timing combination may synergistically enhance motor function remodeling by dynamically regulating the inhibitory balance between the two hemispheres and offer fresh evidence for optimizing therapeutic parameters of rTMS in conjunction with CIMT. In an analytical randomized controlled study, 45 hemiplegic youngsters were assigned to one of five groups: CIMT, 1 Hz daily rTMS, neither group, or both groups. Researchers conducted rTMS for 14 days, focusing on the contralesional hemisphere at the primary motor region as the stimulation site. The rTMS was administered for 14 days, and the COPM scale was utilized to assess the quality of life in the rTMS combined with the CIMT group at the 6 months mark. This demonstrated that the two interventions were more effective in conjunction, enhancing the children’s engagement in functional activities and improving their quality of life, surpassing the outcomes of the individual therapies administered separately (Kirton et al., 2016).

**TABLE 1:** Comparison of parametric regimens and efficacy of combination therapies.

References	Patient	Group	rTMS parameter	CIMT program	Evaluation index	Result
Kakuda et al., 2011	Number: 52 Age: 57 ± 13 years Illness: stroke and had upper limb hemiparesis State: after onset 50 ± 33 months	Stage 3 group (*n* = 13) Stage 4 group (*n* = 20) Stage 5 group (*n* = 19) (Based on the Brunnstrom stage for hand-fingers at admission)	Quantity:1,200 pulses Intensity:90% of motor threshold of FDI muscle Frequency:1 Hz Target: the skull of the contralesional hemisphere Course of treatment: Twenty-two 20 min treatments in 15 days.	Interventions: A 60 min one-on-one training and a 60 min self-training. Course of treatment: Twenty-two 120 min treatments in 15 days.	FMA (Fugl-Meyer Assessment) WMFT (Wolf Motor Function Test)	FMA: Stage 4 group>Stage 3 group>Stage 5 group WMFT: Stage 4 group>Stage 5 group>Stage 3 group
Kakuda et al., 2012	Number: 204 Age: 58.5 ± 13.4 years Illness:Stroke and had upper limb hemiparesis State: After onset 5.0 ± 4.5 years	–	Quantity: 1,200 pulses Intensity: 90% of motor threshold of FDI muscle Frequency: 1 Hz Target: the contralesional hemisphere over the primary motor area Course of treatment: Twenty-two 20 min treatments in 15 days.	Interventions: A 60 min one-on-one training and a 60 min self-training. Course of treatment: Twenty-two 120 min treatments in 15 days.	FMA (Fugl-Meyer Assessment) WMFT (Wolf Motor Function Test)	FMA↑ (Median FMA score of 47 improved to 51) (*P* < 0.001) WMFT↑ (Median WMFT log performance time 3.23 shortened to 2.51) (*P* < 0.001)
Gillick et al., 2014	Number: 19 Age: 10.8 ± 2.8 years Illness: congenital hemiparesis State: manual ability classification scale levels I–III	Control group: rTMS–/CIMT+ (*n* = 9) Intervention group: rTMS+/CIMT+ (*n* = 10)	Quantity: 600 priming pulses and 600 low frequency pulses Intensity: 90% of motor threshold of FDI muscle Frequency: 10 min 1 Hz and 10 min 6 Hz alternately Target: the contralesional hemisphere over the primary motor area Course of treatment: Five 20 min treatments in 14 days.	Interventions: continuous long-arm casting and skin-check sessions Course of treatment: 13 days	AHA (Assisting Hand Assessment) COPM (Canadian Occupational Performance Measure)	AHA: Intervention group>Control group (*P* = 0.007). COPM: no significant differences in the COPM were found.
Kirton et al., 2016	Number: 45 Age: 6∼19 years Illness: MRI-confirmed unilateral perinatal ischemic stroke State: Symptomatic hemiparesis (Including perceived functional limitations by child and parent)	Control group: rTMS–/CIMT– (*n* = 12) rTMS–/CIMT+ (*n* = 11) rTMS+/CIMT– (*n* = 10) Intervention group: rTMS+/CIMT+ (*n* = 12)	Quantity: 1,200 pulses Intensity: 90% of motor threshold of FDI muscle Frequency: 1 Hz Target: the contralesional hemisphere over the primary motor area Course of treatment: Five 20 min treatments in 14 days.	Interventions: 2 h of direct, one-onone motor learning therapy each day, Interventions were individualized to the specific goals of each child. Course of treatment: 14 days	AHA (Assisting Hand Assessment) COPM (Canadian Occupational Performance Measure)	At the AHA and COPM assessments at 6 months, the rTMS+/CIMT+ group had the greatest benefit. (*P* = 0.0004)
Kuthiala et al., 2020	Number: 60 Age: 44.6 ± 10.4 years Illness: chronic stroke State: after onset 24 ± 12 months	Control group: rTMS–/CIMT+ (*n* = 30) Intervention group: rTMS+/CIMT+ (*n* = 30)	Quantity: 2,000 pulses Intensity: 110% of motor threshold of FDI muscle Frequency: 10 Hz Target: dorso lateral prefrontal cortex Course of treatment: a total 15 treatments were given 5 days/week for 3 weeks.	Interventions: patients were given CIMT by a neurophysical therapist for 1 h. Course of treatment: total 3 weeks (15 treatments)	BOLD (FMRI) FM (Fugl Meyer) MRS (Barthel Index modified Rankin Scales)	FM: Intervention group>Control group (At 3 weeks and 3 months) BOLD: the BOLD cluster activation was compared between two groups; there was increase in the number of clusters found in Intervention group.
Han, 2022	Number: 90 Age: 40∼70 years Illness: stroke and had upper limb hemiparesis (Including 60 patients with cerebral infarction and 30 patients with cerebral hemorrhage) State: after onset 1∼6 months	Control group: rTMS+/CIMT– (*n* = 30) rTMS–/CIMT+ (*n* = 30) Intervention group: rTMS+ CIMT (*n* = 30)	Quantity: 1,200 pulses Intensity: 90% of motor threshold of FDI muscle Frequency: 1 Hz Target: the contralesional hemisphere over the primary motor area Course of treatment: a total 20 treatments were given 5 days/week for 4 weeks.	Interventions: restriction of the use of the healthy limb, forcing the patient to use the affected upper limb. Course of treatment: once a day, 5 days a week for 4 weeks.	WMFT (Wolf Motor Function Test) FMA-UE (Fugl-Meyer assessment-upper extremity) UEFT (Upper extremity function test) MBI (modified Barthel index)	Pre-treatment: there were no significant differences in WMFT/FMA-UE/UEFT/MBI scores in three groups. (*P*>0.05) Post-treatment: WMFT/FMA-UE/UEFT/MBI scores were significantly higher in all three groups. (*P* < 0.01) WMFT Intervention group group>CIMT group>rTMS group (*P* < 0.05) FMA-UE Intervention group group>CIMT group>rTMS group (*P* < 0.05) UEFT Intervention group group>CIMT group>rTMS group (*P* < 0.05) MBI Intervention group group>CIMT group>rTMS group (*P* < 0.05)
Gupta et al., 2023	Number: 46 Age: 5∼18 years Illness: gross motor function State: unilateral cerebral palsy classification system (GMFCS) stage 1∼4 and manual ability classification stage (MACS) stage 1∼3	Control group: rTMS–/CIMT+ (*n* = 23) Intervention group: rTMS+/CIMT+ (*n* = 23)	Quantity: 600 priming pulses and 600 low frequency pulse Intensity: 90% of motor threshold of FDI muscle Frequency: 10 min 1 Hz and 10 min 6 Hz alternately Target: the contralesional hemisphere over the primary motor area Course of treatment: Ten 20 minute treatments in 28 days.	Interventions: the mCIMT sessions were structured and individualized for each child. Course of treatment: 56 h over 4 weeks with a minimum of 2 h per treatment.	QUEST (Quality of Upper Extremity Skills Test)	QUEST: Intervention group>Control group
Xue et al., 2024	Number: 80 Age: 44∼69 years Illness: acute Ischemic Stroke State: after onset 14∼30 day	Control group: rTMS–/CIMT+ (*n* = 40) Intervention group: rTMS+/CIMT+ (*n* = 40)	Quantity: 1,200 pulses Intensity: 100% of motor threshold of FDI muscle Frequency: 3 Hz Target: head area Course of treatment: once every 2 days, 20 min/time, lasting for 1 month.	Interventions: mirror-image exercise therapy and resistance training. Course of treatment: once a day, 4 days a week for 1 month.	NIHSS (National Institutes of Health Stroke Scale) BI (Barthel Index) BBS (Berg Balance Scale) FMA (Fugl-Meyer Motor Assessment)	Pre-treatment: there were no significant differences in NHISS/BI/BBS/FMA scores between two groups. (*P* > 0.05). Post-treatment: NHISS Control group>Intervention group (*P* < 0.05) BI Intervention group>Control group (*P* < 0.05) BBS Intervention group>Control group (*P* < 0.05) FMA (Upper and lower limbs) Intervention group>Control group (*P* < 0.05)

Significant advancements have recently been achieved in the rehabilitation of individuals with upper limb hemiplegia following a stroke. Neuromodulation techniques and behavioral training synergistically yield significant advantages. Several astute researchers integrated rTMS with CIMT. The frequency employed was 10 Hz, representing 110% of the motor threshold of the first dorsal interosseous muscle. The dorsolateral prefrontal cortex served as the target for rTMS stimulation. The group exhibited a more pronounced enhancement in Fugl-Meyer motor function scale (FM) scores 3 weeks post-intervention in comparison to the CIMT group alone. This enhancement was also observed at the 3 weeks and 3 months follow-ups post-treatment. Functional magnetic resonance imaging (fMRI) revealed significantly greater activation clusters of blood oxygen level-dependent (BOLD) signaling in the brains of patients in the experimental group. This indicates that high-frequency rTMS may enhance the functional connection of the DLPFC with the motor cortex. This underscores the significance of integrating central modulation with peripheral motor training in a comprehensive rehabilitation approach for chronic phase stroke patients, demonstrating clinical applicability (Kuthiala et al., 2020). A study involving 90 stroke patients (60 with cerebral infarction and 30 with cerebral hemorrhage) demonstrated that a 4 weeks intervention protocol utilizing 1 Hz rTMS targeting the contralesional hemisphere over the primary motor area, in conjunction with CIMT, resulted in enhanced scores on the Wolf Motor Function Test (WMFT), Fugl-Meyer Assessment-Upper Extremity (FMA-UE), upper extremity function test (UEFT), and modified Barthel index (MBI). This resulted from a substantial synergistic effect. The study results demonstrated that the mean scores of the combined treatment group across the four scales were significantly elevated compared to the control group receiving only forced exercise or single rTMS (*p* < 0.05), thereby substantiating the clinical benefits of the central-peripheral synergistic intervention model in the rehabilitation of patients with varying degrees of stroke (Han, 2022). A study on pediatric cerebral palsy (CP) by Gupta et al. (2023) sought to apply rTMS in conjunction with modified CIMT (mCIMT) at 6 Hz, focusing on the contralesional hemisphere above the major motor region. The Quality of Upper Extremity Skills Test (QUEST) verified that the mean change in the QUEST total score for the intervention group was considerably greater than that of the control group at 4 weeks, demonstrating that 6 Hz rTMS paired with CIMT is both safe and practical. The group’s mean alteration in the QUEST score significantly exceeded that of the control group. This indicates that the application of rTMS at 6 Hz in conjunction with CIMT is both safe and feasible. This provides a framework for identifying the optimal treatment plan by modulating the intensity of the CIMT intervention and the parameters of task-oriented training. Broadening the research scope is a significant aspect of the present advancements. Xue et al. (2024) has achieved a significant advancement in stroke rehabilitation. The study used 3 Hz rTMS, with a stimulation intensity set at 100% of the movement threshold for the first dorsal interosseous muscle, targeting the cranial region. It employs an innovative CIMT program in conjunction with a multidimensional assessment system [National Institutes of Health Stroke Scale (NIHSS), Barthel Index (BI), Berg Balance Scale (BBS), and Fugl-Meyer Motor Function Assessment Scale (FMA)] to systematically evaluate the clinical efficacy of the combined intervention. One month later, the study’s results indicated that, with the exception of the NIHSS, the combined therapy group had considerably superior BI, BBS, and FMA scores compared to the control group, which got only behavioral training. The FMA subgroup analysis indicated that the enhancement of motor function in both upper and lower limbs occurred simultaneously, which constituted an additional advantage. This was the inaugural instance in which the combined therapy was systematically demonstrated to enhance the overall motor function of stroke patients. The combination therapy may exert a comprehensive regulatory influence via the trans-hemispheric neuronal network. The findings indicate that the combination therapy may exert a worldwide regulatory influence via the trans-hemispheric neural network. This provides us with data that can be utilized in the future to determine the optimal therapeutic parameters for combination therapy. These insights have enabled the development of neuroplasticity-based models for integrated central and peripheral therapies. In the future, researchers will concentrate on identifying the precise location of optimal stimulation parameters.

## 5 Limitations

Historically, rTMS has been shown to be safe and effective as a rehabilitation therapy, and its safety requires special attention to the following factors: metal implants in the patient’s body need to be kept at a safe distance to avoid magnetic field interference, people with a history of epilepsy or those who are taking medications to lower the epileptic threshold need to be carefully assessed for risk, and parameter settings need to be in accordance with international safety guidelines in order to reduce the probability of epileptic seizure triggers (Luo, 2020); and CIMT has also proved to be safe for treating limb dysfunction after a stroke. CIMT has also been shown to be safe in the treatment of post-stroke limb dysfunction, with safety considerations including avoidance of overexertion or strain of the affected limb, attention to the risk of skin compression injuries, and its non-invasive nature is more easily accepted by patients and can effectively facilitate their participation in daily activities (Zhang et al., 2023). Nonetheless, low-frequency rTMS combo therapy is more tolerable for patients, alleviating pain, muscle spasms, and discomfort compared to high-frequency rTMS (Kaur et al., 2019). A recent randomized controlled experiment with blinded assessors demonstrated that children with unilateral cerebral palsy can benefit from low-frequency rTMS in conjunction with CIMT. This therapy is also secure for neurorehabilitation (Wu et al., 2022). A study including 45 stroke patients has shown that an intervention utilizing 10 Hz rTMS in conjunction with exercise treatment is safe (Wang et al., 2020). Lomarev et al. (2007) evaluated the safety of 20 and 25 Hz rTMS applied to the motor cortex of the afflicted hemisphere by assessing the electrical activity of the hand and arm muscles. They observed transient, significant alterations in the EMG, indicating that high-frequency rTMS is safe for healthy volunteers but may increase the risk of seizures in individuals with a history of stroke.

## 6 Perspectives and future direction

Numerous clinical trials have demonstrated that rTMS in conjunction with CIMT therapy is both safe and effective. Significant opportunities remain for research and public understanding regarding the rehabilitation of mobility problems following a stroke. This is an innovative method for post-stroke rehabilitation that will provide fresh insights for future recovery strategies. However, this study presents some issues: (1) The mechanism of action of the combination therapy is not elucidated; (2) additional studies employing standardized combination protocols are necessary to evaluate the efficacy of the treatment; (3) the sample size for this combination therapy is limited; and (4) the optimal stimulation parameters, such as intensity, frequency, therapeutic target, duration, and interval, remain ambiguous. Future research may investigate the standardization of combination therapy, particularly regarding stimulation parameters. Diverse assessment criteria employed for combination therapy complicate the comparison of findings across various research. It is hoped that assessment standards might be standardized in the future. As rehabilitation technology undergoes continuous iterative updates and the research team matures, further validation and enhancement of synergistic rehabilitation therapy techniques will more effectively restore the functional abilities of patients with post-stroke movement disorders.
